# A rapid, low-cost, and microfluidic chip-based system for parallel identification of multiple pathogens related to clinical pneumonia

**DOI:** 10.1038/s41598-017-06739-2

**Published:** 2017-07-25

**Authors:** Guoliang Huang, Qin Huang, Lan Xie, Guangxin Xiang, Lei Wang, Hui Xu, Li Ma, Xianbo Luo, Juan Xin, Xinying Zhou, Xiangyu Jin, Lei Zhang

**Affiliations:** 1 Department of Biomedical Engineering, the School of Medicine, Tsinghua University, Beijing, 100084 China; 2 National Engineering Research Center for Beijing Biochip Technology, Beijing, 102206 China

## Abstract

An air-insulated microfluidic chip was designed for the automatic centrifugal distribution of samples to 24-test cells, enabling the parallel identification of multiple clinical pneumonia-related pathogens in 1.45-μL reactions without cross-contamination in 45 min. A portable nucleic acid analyzer that integrates mechanical, confocal optical, electronic, and software functions was also developed to collect fluorescence data in a Ø3 mm imaging field near the optical diffraction limit for highly sensitive fluorescence detection of nucleic acid amplification in real time. This microfluidic chip-based portable nucleic acid analyzer could detect low abundance nucleic acids present at as few as 10 copies. In a blinded experiment, specific identification of *Mycoplasma pneumoniae*, *Staphylococcus aureus*, and methicillin-resistant *S. aureus* was achieved with 229 clinical patient sputum samples. The total coincidence rate of our system and traditional RT-PCR with an ABI 7500 was 99.56%. Four samples accounting for the 0.44% inconformity were retested by gene sequencing, revealing that our system reported the correct results. This novel microfluidic chip-based detection system is cost-effective, rapid, sensitive, specific, and has a relatively high throughput for parallel identification, which is especially suitable for resource-limited facilities/areas and point-of-care testing.

## Introduction

Pneumonia is one of the most serious infectious diseases with high morbidity and mortality. In 2015, pneumonia killed an estimated 922,000 children under 5 years old, accounting for 15% of all deaths of children in that cohort (www.who.int). Children below the age of 5 and seniors over the age of 65 are more susceptible to developing severe pneumonia due to their weaker immune systems^1, 2^. Early and appropriate antibiotic administration is crucial for the prognosis of pneumonia. However, the emergence of drug-resistant bacteria complicates the empirical treatment of pneumonia. For example, penicillin resistance has become widespread, and patients with methicillin-resistant *Staphylococcus aureus* (MRSA) pneumonia have a higher risk of treatment failure^3^.

Rapid and accurate identification of pathogens and their resistance to antibiotics is critical for timely diagnosis and antibiotics choice, but the traditional culture-based method to determine this information is inadequate in many respects. Although regarded as the gold standard, culturing usually takes 72–96 h, with a relatively high false negative rate, especially for pathogens that are difficult to culture, and requires a certain amount of sample to start the culturing process. Due to these disadvantages, nucleic acid-based testing is becoming increasingly popular because it is direct, sensitive, and rapid. The most powerful method of this kind is PCR, but even it has drawbacks. These include the requirement for a thermocycler, which enables rapid heating/cooling temperature cycles but makes the equipment expensive and not suitable for resource-limited facilities and areas^4^. Moreover, because pathogenic species capable of causing pneumonia are numerous, it would be more economical if all suspected pathogens could be accurately identified in a single assay. This is also challenging for PCR because even multiplex PCR cannot simultaneously detect more than four to five targets.

Efforts to overcome the limitations of PCR have resulted in other methods, such as isothermal amplification techniques, that do not require thermal cycling but instead rely on enzymatic activities for DNA/RNA synthesis^5^. Notomi *et al*. report a novel nucleic acid amplification method called loop-mediated isothermal amplification (LAMP), which is capable of amplifying DNA under isothermal conditions with remarkable specificity, efficiency, and speed^6^. The assay is simple and inexpensive because minimal laboratory infrastructure is required, and it has recently been used to diagnose various infectious diseases^7, 8^. Yoshino *et al*. report a LAMP assay using a set of primers targeting the syndecan 1 repetitive element of the *Mycoplasma pneumoniae (Mpn)* genome^9^. Gotoh *et al*. report a highly sensitive and specific LAMP assay for *Mpn* detection^10^.

Microfluidic chips are a promising platform for pathogen detection. These so-called “micro total analysis systems (μTAS)” or labs-on-a-chip (LOC) have gained in popularity due to their flexibility for automation, integration, miniaturization, and multiplexing. Pathogen detection based on microfluidic chips also has many other advantages. Because the reaction chambers are usually on the micro- or nano-scale, the devices can be miniaturized and portable, and are therefore suitable for point-of-care testing. LOC technology allows for the integration of sample preparation, amplification, and signal detection, which reduces the time need to generate results. The high throughput and low consumption of sample and reagents make the technology flexible and relatively cost effective. Nucleic acid-based microfluidic pathogen detection has been achieved for the detection of bacteria, viruses, and fungi^11–13^. Indeed, commercial chip-based pathogen detection systems are now emerging^14^. However, most of these systems utilize PCR or real-time PCR for amplification.

In recent years, integrated microfluidic LAMP systems have been reported, and different detection methods have been developed^15–18^. In most studies, centrifugal pumping is used and accompanied by specially fabricated valves, such as capillary, hydrophobic, sacrificial^19^, and burst valves^20^. However, the centrifugal pressure must be equal to or greater than the surface tension pressure of the valves, or the fluid will begin to flow once again. A valve-less chip would make the device simpler to use and the fluid more stable. Moreover, it is important to validate the performance of a system with real clinical samples instead of cultured strains.

**Figure 1:**
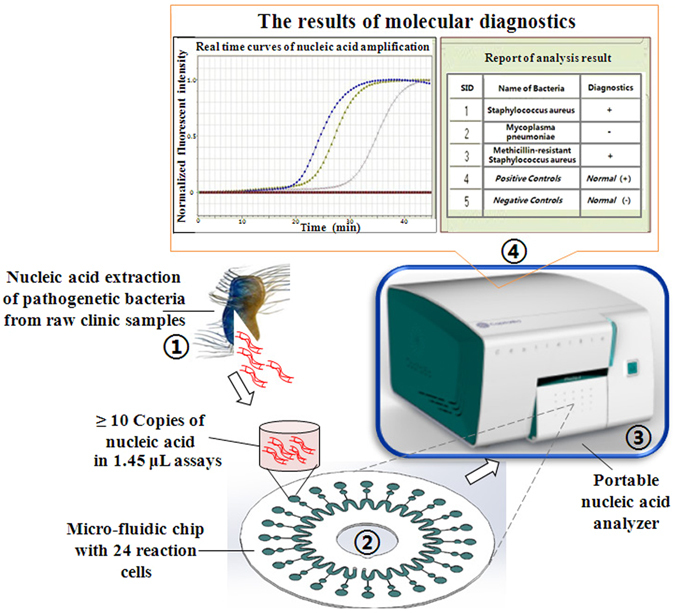
Parallel identification of multiple pathogens using the microfluidic chip-based portable nucleic acid analyzer.

In this study, we established a valve-less, air-insulated microfluidic chip for the identification of multiple pneumonia-related pathogens as shown in Fig. 1. A custom microfluidic chip capable of simultaneously detecting 24 species of pneumonia-related pathogens (without cross-contamination) in 45 min and a matched device with an integrated detection system were developed. The device had high sensitivity (detecting as few as 10 copies of nucleic acid) and utilized low-cost 1.45-μL reactions, making it suitable for point-of-care testing. We validated the performance of the new system via a blind assessment of 229 clinical samples for three pathogens: *Mpn*, *S. aureus (Sau)*, and a MRSA strain. The total coincidence rate of the test device and the control device (*i.e*., an ABI 7500) was 99.56%. Four samples exhibited different results between the two platforms. Sequencing of the four samples suggested that results from our platform were in better accordance with the sequencing results than those from the ABI 7500. Thus, we believe this novel device is even more suitable than the ABI7500 for pneumonia-related pathogen detection, especially for samples with extremely low concentrations.

## Results

### Microfluidic chip for parallel identification of multiple pathogens

**Figure 2:**
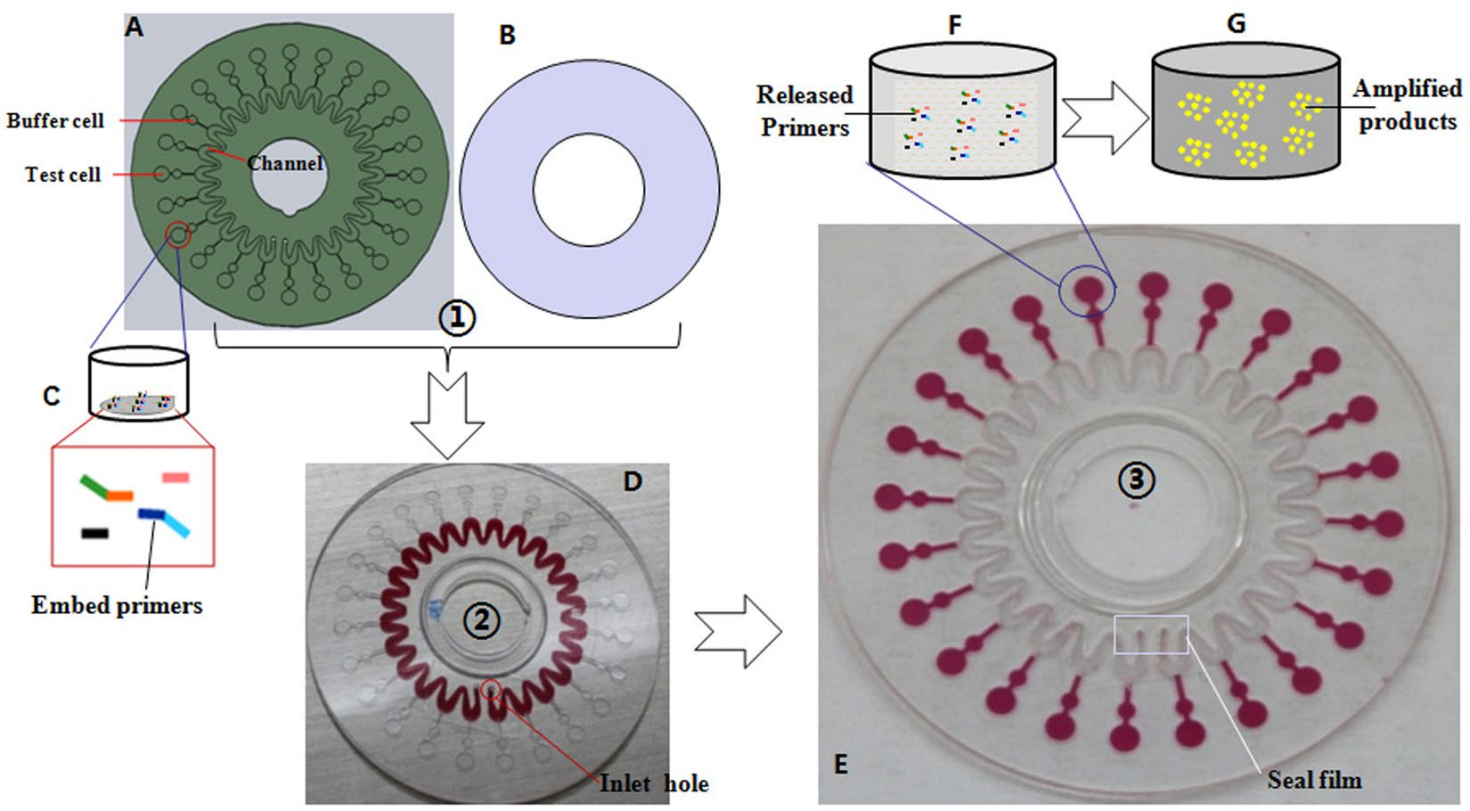
Structure of the microfluidic chip and method for the parallel identification of multiple pathogens. (**A**) Basement of the microfluidic chip. (**B**) Cover of the microfluidic chip. (**C**) Six primers embedded together at the bottom of one test cell using low melting point Sepharose CL-4B. (**D**) The mixture of the prepared DNA sample and isothermal nucleic acid amplification reactants is injected into the microfluidic chip *via* the inlet hole using a pipette. (**E**) The mixtures after being centrifuged at 5000 rpm. (**F**) Six primers released at >50 °C. (**G**) The fluorescent marker EvaGreen bound to the amplified products as nucleic acid amplification occurred at 65 °C.

The microfluidic chip for parallel identification of multiple pathogens was developed *via* the following steps:

1. Design of the novel air-insulated microfluidic chip. A novel valve-less, air-insulated microfluidic chip was designed for the parallel identification of multiple pathogens as illustrated in Fig. 2(A) and (B). The basement and the cover were both 60 mm in diameter and 0.6 mm in thickness. There are 24 test cells and the same number of buffer cells on the basement. The test cells are 3 mm in diameter, with a depth of 0.2 mm and volume of 1.45 μL. The buffer cells are 1.5 mm in diameter and 0.2 mm deep. All test and buffer cells were connected to an approximate sine-type channel *via* 24 pipes (0.2 mm (width) × 0.1 mm (depth)). This approximate sine-type channel consisted of 24 U-type units; each U type unit was connected to a test cell and a buffer cell. The buffer cell was used to compensate for the volume error from the fabrication of the microfluidic chip and the sampling process by pipettes, and it ensured that all liquid in the 24 U-type units (as shown in Fig. 2(D)) could be centrifuged without leaving residue to completely fill the 24 test cells as in Fig. 2(E). This also created an air insulation zone among the 24 test cells to prevent cross contamination.
2. Fabrication of the microfluidic chip. The microfluidic chip was fabricated with PC materials *via* precision injection molding, resulting in a smooth surface with a roughness < 10 μm in all test cells, buffer cells, sine-type channels, and pipes, which is very important to reduce nonspecific adsorption of reagents to the surface of the chip. After one 70% ethanol rinse followed by two water washes, the microfluidic chip can be used. Six primers designed to identify one pathogen each were embedded together at the bottom of each test cell using low melting point Sepharose CL-4B, as shown in Fig. 2(C). After all primers for different pathogens were designed (Table 1), as well as the positive and negative controls, they were independently embedded in different test cells of the basement (Fig. 2(A)). The cover (Fig. 2(B)) was tightly adhered to the basement using double-sided adhesive film with a thickness of 5–6 μm under 100 Kg pressure, and thus, the microfluidic chip was ultimately obtained.
3. Injection of the prepared DNA sample and isothermal nucleic acid amplification reactants into the microfluidic chip. First, the prepared DNA sample and isothermal nucleic acid amplification reactants were evenly mixed in a 1-mL microcentrifuge tube. Then, the mixture was injected into the microfluidic chip from the inlet hole using a pipette as shown in Fig. 2(D). After the inlet hole was sealed with a 5 × 10 mm seal film, the microfluidic chip was centrifuged at 5000 rpm for 10 s, mixing the prepared DNA sample and the isothermal nucleic acid amplification reactants in the test cells as shown in Fig. 2(E).
4. Use of the microfluidic chip for parallel identification of multiple pathogens. The Sepharose CL-4B at the bottom of the test cells (Fig. 1) is dissolved when the device heats to 50 °C, allowing all primers to be released into the mixtures of the prepared DNA sample and the isothermal nucleic acid amplification reactants as in Fig. 2(F). Then, nucleic acid amplification occurs at 65 °C, and the amplified products of the specific nucleic acid sequence are continuously generated. At the same time, the fluorescent marker EvaGreen automatically binds to these amplified products, enabling real-time fluorescent signal detection by the portable analyzer for parallel identification of multiple pathogens as shown in Fig. 2(G).

**Table 1:** Sequences of the primer sets used in this study.

Pathogenic	Primers	Sequence (5′-3′)
*Staphylococcus aureus*	Sau-F3	GTGCCTTTACAGATAGCATG
	Sau-B3	GAAAAAGTGTACGAGTTCTTGA
	Sau-FIP	GTTTCATAACCTTCAGCAAGCTTTCCATACAGTCATTTCACGCA
	Sau-BIP	GAGGTCATTGCAGCTTGCTTACTTCGATCACTGGACCGCG
	Sau-LF	AACTCATAGTGGCCAACA
	Sau-LB	GTACCTGTTATGAAAGTGTTCA
MRSA	MRSA-F3	TTATGGCTCAGGTACTGCT
	MRSA-B3	TTTTGTTATTTAACCCAATCATTGC
	MRSA-FIP	ATTCTTCGTTACTCATGCCATACATGTGAATTATTAGCACTTGTAAGCAC
	MRSA-BIP	AACCGAAGATAAAAAAGAACCTCTGAATATTTTTTGAGTTGAACCTGGTG
	MRSA-LF	AATGGATAGACGTCATATGAAGGT
	MRSA-LB	CTCAACAAGTTCCAGATTACAACTT
*Mycoplasma Pneumonia*	Mpn-F3	CTCACCGTAGTGGGACA
	Mpn-B3	GCCCCGGGATTTTCACC
	Mpn-FIP	CGTCAGGGCGGGTGTAGCTCTTCACAAGTACCACCACGAC
	Mpn-BIP	TGCGCCACACCAATGCCATGGGAGGGAGGAAAAGCT
	Mpn-LF	ATTGCTGGCGCTTGAGC
	Mpn-LB	CGCGCTTAACCCCGTGA

### Oligonucleotide primers for parallel identification of three pneumonia pathogens

We next attempted to determine whether the device could be used for molecular diagnostics to identify multiple pathogens in parallel. In a proof-of-concept experiment, three groups of oligonucleotide primers were designed for the isothermal amplification assay according to the sequences of the *femA* gene of *Sau* (Gen-Bank accession no. BA000033), *mecA* gene of MRSA (Gen-Bank accession no. CP000046), and *SeqA* gene of *Mpn* (Gen-Bank accession no. CP002077) using Primer Explorer version 4 (https://primerexplorer.jp/elamp4.0.0/index.html). Details concerning the primers are listed in Table 1. All of the primers were synthesized by Invitrogen (Shanghai, China). The F3 and B3 primer pair also worked as sequencing primers to confirm the presence of the target genes.

### Portable nucleic acid analyzer for real-time fluorescence detection and molecular diagnostics on a microfluidic chip

**Figure 3:**
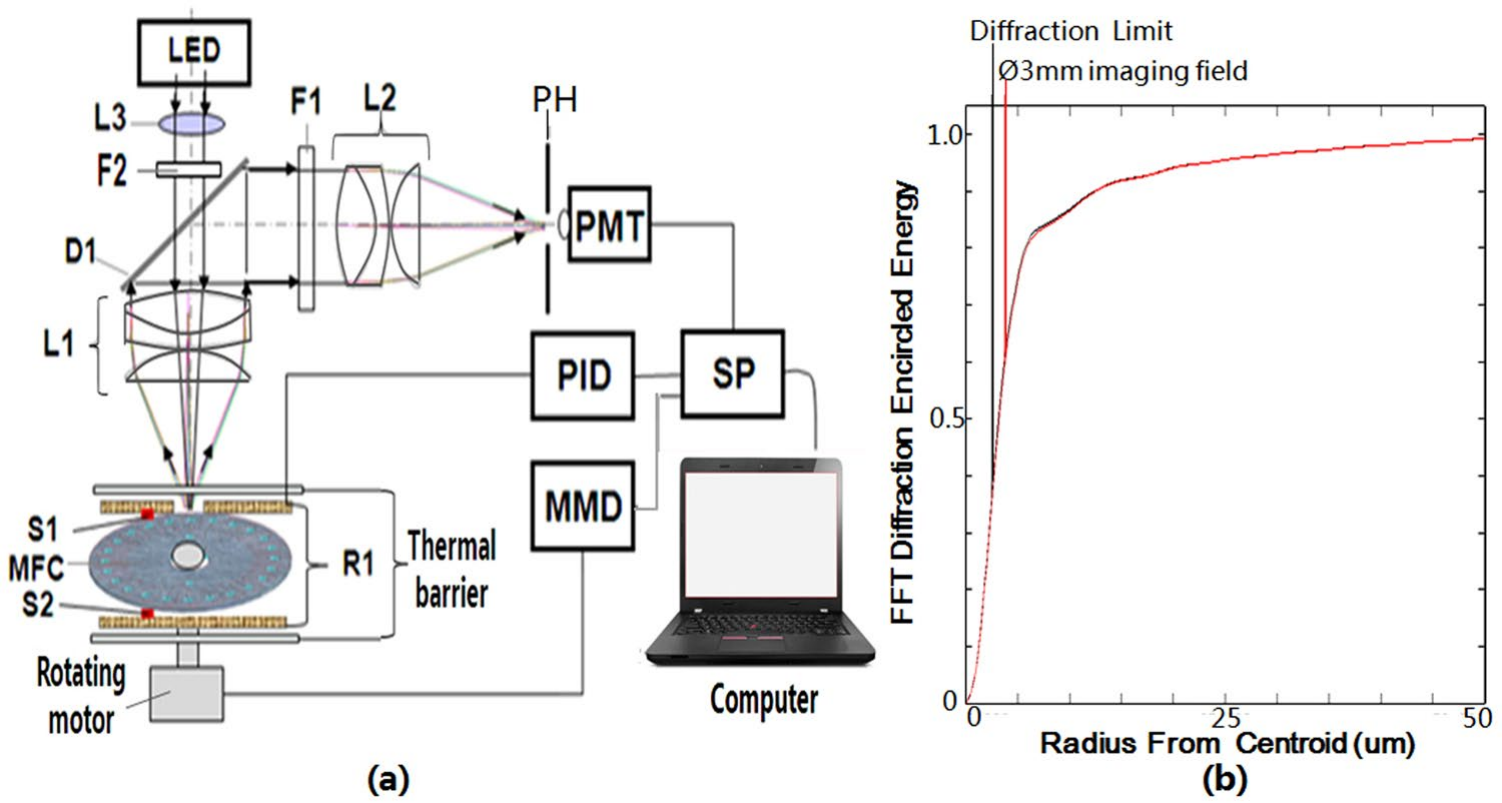
Portable nucleic acid analyzer for parallel identification of multiple pathogens. (**a**) Principle structure of the portable nucleic acid analyzer. (**b**) Diagram of a diffraction energy simulation.

A portable nucleic acid analyzer was developed to perform sequence-specific molecular diagnostics on the microfluidic chip as shown in Fig. 3.

Figure 3(a) depicts the structure of the portable nucleic acid analyzer. The confocal optical detection unit of the portable nucleic acid analyzer consists of the excited optical path and the fluorescent collection path. In the excited optical path, the excited light from a 1 W blue LED is first collimated by an aspherical lens L3 with a focal length of 20 mm, secondarily filtered by the band pass filter F2 (470 nm central wavelength and 30 nm band width), and then transmitted to the dichroic mirror D1 (high transmission to 400–500 nm light and reflection to 500–600 nm light). Finally, it is focused into the test cell of the microfluidic chip (MFC) by the objective L1 with a focal length of 22 mm and excites the amplified products bound to the fluorescent marker EvaGreen in the test cell, yielding a real-time fluorescent signal. In the fluorescent collection path, the fluorescent signal from the test cell is first collected in real time by the objective L1, reflected by the dichroic mirror D1, and then filtered by the band pass filter F1 (530 nm central wavelength and 30 nm band width). Next, it is focused by the imaging lens L2 with the same focal length as L1 onto the pinhole PH to filter the farraginous light from the environment and the off-focus chip material. Finally, it is detected by the photomultiplier tube (PMT, Hammatsu, Japan). A rotary motor drives the microfluidic chip rotation and enables all test cells to be detected in a period of 30 s by the photomultiplier tube. The real-time fluorescent signal is then transferred by the processor SP and shown on the computer. The heater R1 is kept at a 0.25-mm gap to the microfluidic chip to enable fast, even, two-sided heating. The temperature controller (PID) is used to control the heater R1 with an accuracy of 0.1 °C and a heating rate of 1 °Cs^−1^ by means of the feedback from a temperature sensor (S1 and S2). A multiple-axis moving driver (MMD) is used to control the rotary motor at an angular accuracy of 0.01°. Figure 3(b) shows the Fast Fourier Transformation (FFT) diffraction encircled energy for the confocal optical detection of the portable nucleic acid analyzer, which indicates that the fluorescence collection in a Ø3-mm imaging field is near the optical diffraction limit and has an adequate sensitivity to detect the amplified products of the trace nucleic acid in the test cell of the microfluidic chip.

A photo of the portable nucleic acid analyzer and the main process to identify multiple pathogens in parallel using the analyzer is shown in Fig. 1. Trace nucleic acids from pathogenic bacteria were first extracted from the sputum of inpatients and outpatients and then mixed with the isothermal nucleic acid amplification reagents in a 1-mL microcentrifuge tube. Next, the mixture was injected into the microfluidic chip and centrifuged at 5000 rpm for 10 s. Then, the microfluidic chip was placed into the portable nucleic acid analyzer for incubation at 65 °C for 45 min. The fluorescence signals of the amplified products were detected, and the nucleic acid amplification curves were displayed in real time.

EvaGreen was used to follow the nucleic acid amplification in real time. In general, free EvaGreen does not fluorescence, but when it binds to double-stranded DNA (*e.g*., nucleic acid amplification products), it can be excited by blue light and emit a fluorescent signal. Free EvaGreen was mixed into the isothermal amplification assays. Therefore, when the nucleic acid amplification is performed in the presence of DNA from a pathogen, amplification products are continually made, and EvaGreen binds to them. The portable nucleic acid analyzer can detect the increase in the fluorescence signal in real time, and exponentially increasing nucleic acid amplification curves are obtained. If no DNA from a pathogen is present, then no nucleic acid amplification occurs, and the free EvaGreen does not emit a fluorescent signal. Thus, the portable nucleic acid analyzer detects no change in fluorescence, and the nucleic acid amplification curves are flat. The results of identifying multiple pathogens in parallel are ultimately reported in the end.

### Limit of detection, reproducibility, and linearity of the portable nucleic acid analyzer-based microfluidic chip

**Figure 4:**
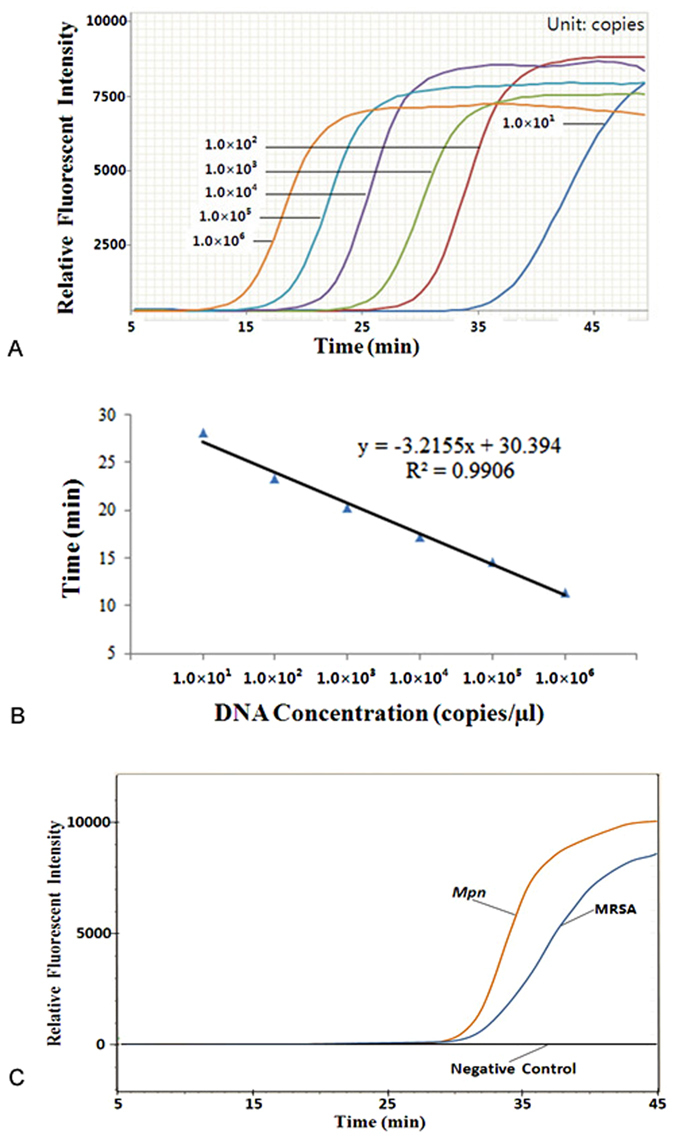
LOD and linearity analysis of the portable nucleic acid analyzer-based microfluidic chip. (**A**) LOD analysis for *Sau*. (**B**) Linearity analysis for *Sau*. (**C**) LOD analysis for *Mpn* and MRSA.

The limit of detection (LOD) of the portable nucleic acid analyzer-based microfluidic chip was assessed using serial dilution of purified *Sau* genomic DNA (gDNA). Six gDNA template samples with different concentrations (1.0 × 10^6^, 1.0 × 10^5^, 1.0 × 10^4^, 1.0 × 10^3^, 1.0 × 10^2^, and 1.0 × 10^1^ copies/µL) were used, and the amplification curves are displayed in Fig. 4. Twenty-four duplicate reactions were performed for every DNA template concentration to evaluate reproducibility. The time to positive value (Tp), which was defined as the time at the second derivative inflexions of the exponential DNA amplification curves, was set to indicate the initiation of the entire system. As shown in Fig. 4, the LOD for isothermal amplification was 10 copies of gDNA for *Sau* for the portable nucleic acid analyzer-based microfluidic chip. The coefficient of variation (CV), defined as the ratio of the standard deviation (SD) to the mean average (CV = SD/AVERAGE × 100%), was used to describe the relative dispersion of the Tp values. A CV value between 0 and 5% indicated good reproducibility, and between 5 and 10% indicated acceptable reproducibility. The CVs of 24 repeated measurements were 5.76%, 2.93%, 1.28%, 1.62%, 2.76%, and 3.6% for DNA concentrations of 1.0 × 10^1^, 1.0 × 10^2^, 1.0 × 10^3^, 1.0 × 10^4^, 1.0 × 10^5^, and 1.0 × 10^6^ copies/µL, respectively, demonstrating the good reproducibility of the portable nucleic acid analyzer. Additionally, the relationship between the Tp value and the amount of bacterial gDNA per reaction was determined, and good linearity was observed (Fig. 4). The square of the regression coefficient (R^2^) was 0.9906 for the portable nucleic acid analyzer. This indicates that the portable nucleic acid analyzer-based microfluidic chip was reliable for the quantification of bacteria by using traditional standard curves, and it was comparable to the ABI 7500. The LODs of MRSA and *Mpn* were next analyzed using the portable nucleic acid analyzer-based microfluidic chip (Fig. 4(C)). The CVs of five repeated measurements at the DNA concentration of 10 copies were 5.2% and 4.95% for MRSA and *Mpn*, respectively.

### Analytical specificity

**Figure 5:**
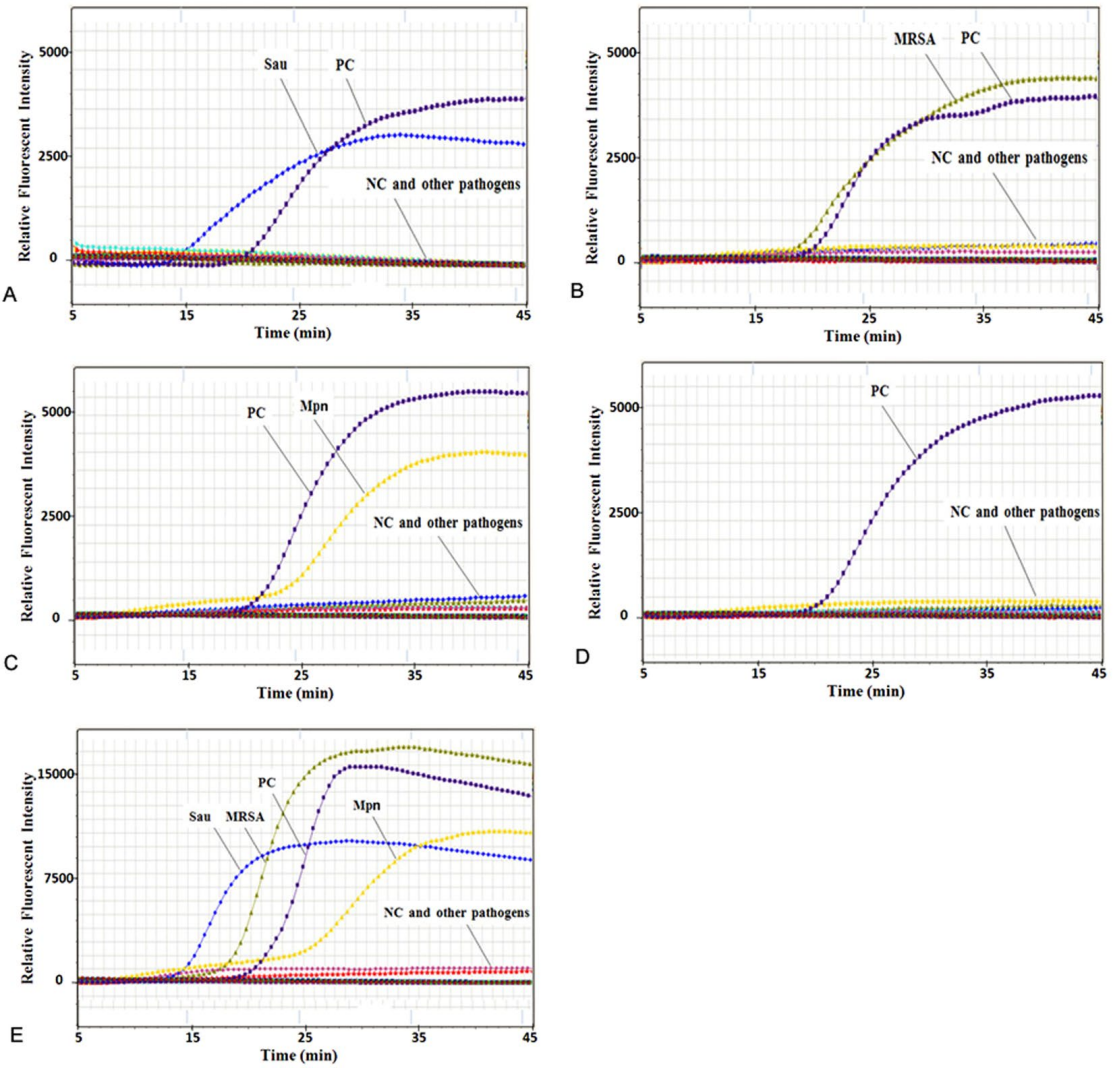
Specificity of the parallel detection assay on the microfluidic chip. (**A**) Detection results of *Sau* nucleic acid. (**B**) Detection results of MRSA nucleic acid. (**C**) Detection results of *Mpn* nucleic acid. (**D**) Detection results of DNA-free H_2_O. (**E**) Detection results of the three target pathogens from a 13-pathogen mixture of gDNA.

The specificity of our system was assessed using prepared gDNA samples from 13 LRT pathogens, including the three target bacteria described above and 10 other interference bacteria: *Escherichia coli (Eco), Pseudomonas aeruginosa (Pae), Streptococcus pneumoniae (Spn), Klebsiella pneumoniae (Kpn), Acinetobacter baumannii (Aba), Stenotrophomonas maltophilia (Sma), Haemophilus influenzae (Hin), Legionella pneumophila (Lpn), Chamydiae pneumonia (Cpn), and Mycobacterium tuberculosis (Mtb)*. All tests were repeated three times. The expected positive signals were recognized by typical sigmoidal amplification curves (Fig. 5), and only the target bacteria displayed positive results. These data revealed that no cross-reactions were introduced by the other 10 species, indicating a high specificity. The negative control (no primers dispensed into the reaction well) displayed no fluorescence throughout the amplification, indicating the low signal background and the absence of contamination.

### Validation of the portable nucleic acid analyzer-based microfluidic chip assay with a blind test of 229 clinic samples

The LRT discharge samples from 229 patients were tested using the microfluidic chip-based portable nucleic acid analyzer and an ABI 7500 instrument along with 25-μL qPCR kits for *Sau*, MRSA, and *Mpn* detection at the same time; the complete results are summarized in Supplemental Info 1. Among the 229 patients, there were 127 children (age <14 years old), 31 young and middle-aged people (age between 15 and 60), and 71 elderly people (age >60 years old). The male:female ratio was 149:80. We found that 225 samples were correctly identified, including 95 positive and 130 negative results. To determine the sensitivity and specificity, we used a four-fold table for calculation, as shown in Table 2, with a detailed description of the calculation process. The agreement between the on-chip LAMP and qPCR assays was assessed using the Kappa (κ) coefficient test.

**Table 2:** Four-fold table to calculate the test results of *Sau*, MRSA, and *Mpn*.

Portable nucleic acid analyzer	ABI 7500					
	Positive			Negative		
	*Sau*	MRSA	*Mpn*	*Sau*	MRSA	*Mpn*
Positive	28	34	33	1	1	1
Negative	0	0	2	200	194	193

The clinical sensitivity and specificity for the three pathogens is summarized in Table 3. They were all >99%, except for the sensitivity of *Mpn* detection, which was 94.3%. The κ was 0.975, 0.975, and 0.941 for *Sau*, MRSA, and *Mpn*, respectively. The *p*-values were <10^−3^, and the *x* ^2^ tests of paired comparison of the enumeration data were 1.000 for all three clinical targets, indicating near-perfect agreement between the two platforms. The total coincidence rates with the ABI 7500 were >98%, indicating high consistency between the two platforms.

**Table 3:** Coincidence rates of the two methods to test for the three bacteria.

Target	Positive coincidence rate (%)	Negative coincidence rate (%)	Total coincidence rate (%)	p(χ^2^)	Kappa value	p(Kappa)
*Sau*	100.0	99.50	99.6	1.000	0.975	0.000
MRSA	100.0	99.49	99.6	1.000	0.975	0.000
*Mpn*	94.3	99.48	98.7	1.000	0.941	0.000

### Discrepancy sample analysis

Compared to the results obtained with the ABI 7500, four clinical samples yielded different test results with our portable nucleic acid analyzer. We reanalyzed the four samples using the two devices, and the results were the same as before (Supplemental Info 1), indicating that the results were stable on both platforms. To further verify the results of these four samples, we sent them for DNA sequencing, which is regarded as the gold standard. The sequencing results were in better accordance with that of the portable nucleic acid analyzer than the ABI 7500 as shown in Table 4. We found that these four samples had a very low bacterial concentration (~1.0 × 10^1^ copies/µL). This indicates that the portable nucleic acid analyzer is superior to the ABI 7500 for analyzing trace samples and is especially powerful for dilute clinical samples.

**Table 4:** Discrepancy sample reanalysis results.

Sample No.	ABI 7500	Reanalysis by ABI 7500	Portable analyzer	Reanalysis by the portable analyzer	Sequencing
R-022	*Mpn*	*Mpn*	MRSA	MRSA	MRSA
R-032	MRSA	MRSA	*Sau*, MRSA	*Sau*, MRSA	*Sau*, MRSA
R-101	Negative	Negative	*Mpn*	*Mpn*	Negative
E-095	*Sau, Mpn*	*Sau, Mpn*	*Sau*	*Sau*	*Sau*

The advanced portable nucleic acid analyzer based on the microfluidic chip was approved as a new medical device (No.20153400580) by the China Food and Drug Administration (CFDA) on April 20, 2015 and supervised as the technology standard of class-III medical setup for its production (CapitalBio Co., Beijing, China) and clinic application.

## Discussion

Although molecular diagnostics is a rapidly developing field^21^, the clinical application of molecular testing is relatively limited. PCR-based pathogen detection is only routinely used in fully equipped first-level hospitals in China, despite the fact that it is sensitive, accurate, and rapid compared to culture-based detection. The high cost of equipment hampers its application in resource-limited facilities, such as community clinics and clinics in villages and towns, especially in developing countries. Further, PCR is also not suitable for point-of-care testing because the equipment is usually bulky. Our newly developed system is cost effective, with no need for a temperature controlling component, and consumes a minimal amount of sample and reagents (1.45-µL reaction volume), which is in contrast to general PCR-based 25-μL reactions in microcentrifuge tubes. The device is small and portable, integrating several functional modules inside. Therefore, it has great potential for application in hospitals and clinics at all levels.

Species are capable of causing pneumonia, including Gram-positive bacteria such as *Spn*, *Sau*, and Group A hemolytic streptococci, as well as Gram-negative bacteria such as *Kpn*, *Hin*, and *Eco*. A typical pneumonia involves pathogens such as *Lpn*, *Mpn*, and *Cpn* ^22^. The throughput of the detection assay is important, and parallel detection of multiple suspected species saves money and time. With a specially designed microfluidic chip, we can simultaneously detect 24 species, which is adequate for common pneumonia pathogen identification. The species to be tested can also be customized by adjusting the primers coated in each well.

MRSA isolates are a serious public health problem that complicate the clinical management of pneumonia^23^. Routine antimicrobial susceptibility testing methods such as disk diffusion, broth microdilution, and agar-based screen tests are very important for clinical decisions. However, the delay between sample acquisition and results report, usually 48–96 h, makes MRSA cross-transmission a potential threat. In this respect, rapid tests for the detection of MRSA (generally based on nucleic acids tests) are a great supplement to conventional methods, especially for screening^24^. Due to their very low turnaround time, nucleic acid amplification tests are extremely suitable to identify patients who are candidates for contact precaution and to decrease nosocomial transmission^25^. In the current work, we only detected the MRSA *mecA* gene with our system, which is the most common gene that mediates methicillin resistance^26^. However, any other gene related to antibiotic resistance can be easily integrated into the platform due to the relatively high throughput of our system.

There are several key points involved in this analyzer. One is a confocal optical detector (Authorized patent ZL200710122151.4, public announcement at www.sipo.gov.cn) developed as the principle structure in Fig. 3(a) (photo of the real setup in Fig. 1). It is capable of highly sensitive fluorescence collection near the optical diffraction limit and reduces the background signal from the material of the microfluidic chip and surroundings. The key features are rotating scanning to decrease photo-bleaching by the excitation light, fast even heating from a resistive film by a thin (0.25 mm thickness) moving air layer of to limit residual denatured DNA during the process of heating to 65 °C, and the moving average filter algorithm (Authorized patent ZL201110113608.1, public announcement at www.sipo.gov.cn) in the software to improve the stability of the real-time fluorescence signal of nucleic acid amplification. All of these are very important for accurate measurements with low sample consumption in 1.45-μL test cells at a high sensitivity (LOD of 10 genomic copies).

With no need for a thermo-cycling process, the LAMP reaction takes <45 min. Pre-treatment of the sample is also simpler compared to that required for conventional PCR because our system has a higher tolerance for impurities in clinical samples. A heating time of 15 min is usually adequate for pre-treatment. Therefore, the overall time from sample pre-treatment to final results is <1.5 h. This is very effective when dealing with critical infectious conditions.

During a blind assessment of 229 clinical samples, we found a coincidence rate of 99.56% with results based on an ABI 7500, and only four samples yielded different results between the two platforms. However, sequencing of the four samples suggested that results from our platform were in better accordance with the sequencing results. When we reanalyzed the four samples, we found that they all had extremely low DNA concentrations, near the detection limitation of 1.0 × 10^1^ copies/µL. This indicates that our portable nucleic acid analyzer is even more suitable than the ABI 7500, especially for trace samples. Taking the results from sequencing as an adjusted reference, the overall sensitivity and specificity of our platform were both better. These applications will further validate the robust performance of our portable nucleic acid analyzer and hopefully lead to its wide acceptance in clinics.

## Methods

### All methods were performed in accordance with relevant guidelines and regulations

#### Standard bacterial strains and DNA template preparation

*Sau* strain ATCC 6538 was obtained from the National Institutes for Food and Drug Control (Beijing, China). The two target genes, *mecA* and *seqA*, were constructed in plasmids at Sangon (Shanghai, China). The DNA templates were extracted and purified using a QIAamp DNA Mini Kit (Qiagen Inc., CA, USA) and quantified with a ND-1000 Spectrophotometer (NanoDrop Technologies, Inc., DE, USA).

#### Sample collection and nucleic acid extraction

A total of 229 lower respiratory tract (LRT) discharge samples were prospectively collected from inpatients and outpatients at the People’s Hospital of Peking University and Beijing Children’s Hospital. The patients were diagnosed with pneumonia, lung infection, acute bronchitis, or acute exacerbation chronic obstructive pulmonary disease. Demographic information was recorded, including clinical diagnosis, age, sex, and sample number. Informed consent was obtained from each participant, and the study was approved by the ethics committee of Tsinghua University. A volume >600 μL of each specimen was stored at −80 °C for further testing. The frozen LRT discharge samples were heated for 15 min before nucleic acid extraction according to biosafety laboratory requirements. gDNA was extracted from samples using a Universal Kit for Bacterial DNA extraction (CapitalBio Co., Beijing, China) according to the manufacturer’s protocol. The prepared DNA samples were immediately stored at −20 °C until use.

#### Isothermal amplification assays

Each 10-μL isothermal nucleic acid amplification assay for pathogen molecular diagnostics consisted of 0.2 μM each of F3 and B3, 1.6 μM each of FIP and BIP, 0.4 μM each of LF and LB, 8 U of *Bst* DNA Polymerase (Large Fragment), 0.1 mM dUTP, 0.4 mM dNTPs (New England Biolabs Ltd., Beverly, USA), 0.5 mg/ml BSA (Fluka Sigma-Aldrich Inc., Missouri, USA), 0.6× EvaGreen (Biotium Inc., California, USA), 0.8 M betaine (Fluka Sigma-Aldrich Inc., Missouri, USA), 6 mM MgSO_4_ (Beijing Chemical Reagents Company, Beijing, China), 0.1 U/mL Uracil-DNA Glycosylase (Fermentas Inc., Burlington, Canada), 10 mM (NH_4_)_2_SO_4_, 20 mM Tris-HCl (pH 8.8 at 25 °C), 10 mM KCl, 0.1% Triton X-100, and 2 μL template DNA. The reaction mixture was incubated at 37 °C for 5 min, then heated to 65 °C and kept at a constant temperature of 65 °C for 40–45 min before ultimately heating to 80 °C for 5 min to terminate the reaction.

#### RT-PCR assays

The prepared DNA samples were then tested by two real-time PCR assays as control methods: the *Sau* and *mecA* genes using *Sau* and MRSA nucleic acid test kits (PCR fluorescence method) (Triplex International Biosciences Co., Ltd, Fujian, China); and *Mpn* using a Mycoplasma Pneumonia nucleic acid test kit (PCR fluorescence method) (ACON Biotech Co., Ltd. Hangzhou, China). The assay procedure, from real-time PCR setup to data acquisition, was completed on the same day using the instructions provided by the kits’ manufacturers. The median fluorescence intensity was generated and automatically analyzed by the ABI 7500 Data Analysis Software GPP version 2.0.4 to establish the presence or absence of targets in each sample.

#### Definition and statistical analyses

The data were analyzed using SPSS version 13.0 (SPSS, Chicago, IL, USA). Differences between the proportions of positive results were compared using NcNemar’s χ^2^ test. The agreement between the on-chip LAMP (as the test platform) and in-tube RT-PCR assays (as the control platform) was assessed using the κ coefficient test. We used a four-fold table to calculate the test results, as shown in Table 5, and the statistical results were analyzed as below:

1. The clinical sensitivity of a target using the test platform = ***A***/(***A*** + ***C***) × 100%;
2. The clinical specificity of a target using the test platform = ***D***/(***B*** + ***D***) × 100%;
3. The total coincidence rate of a target by comparing the test platform with the control platform = (***A*** + ***D***)/(***A*** + ***B*** + ***C*** + ***D***) × 100%;
4. The χ^2^ test of paired comparison of the enumeration data was used to evaluate the agreement of a target between the test platform and the control platform;
5. The κ coefficient test was used to assess the agreement of a target between the test platform and the control platform, *p* < 0.05 was considered to be significant; and
6. The total concordance rate of the test platform and the control platform = ***E***/***F*** × 100%, where ***E*** is the number of samples that had the same result by using the test platform and the control platform, and ***F*** is the total number of clinical samples.

**Table 5:** Four-fold table to calculate the test results.

	Positive (control platform)	Negative (control platform)	Total
Positive (test platform)	*A*	*B*	*A* + *B*
Negative (test platform)	*C*	*D*	*C* + *D*
Total	*A* + *C*	*B* + *D*	*A* + *B* + *C* + *D*

### Availability of materials

The datasets supporting the conclusions of this article are included within the article. The datasets of sequences for the *femA* gene of *Sau, mecA* gene of MRSA, and *seqA* gene of *Mpn* supporting the conclusions of this article can be obtained from their GenBank accessions (https://en.wikipedia.org/wiki/GenBank).

### Ethics approval and consent to participate

Informed and signed consent was obtained and archived for the research performed and publication of the results. The patients consented to clinical molecular diagnostics at People’s Hospital of Peking University and Beijing Children’s Hospital. This study was approved by the ethics committee of Tsinghua University.

## Electronic supplementary material

Supplemental Info 1
