# Supplementary material for: A rapid, low-cost, and microfluidic chip-based system for parallel identification of multiple pathogens related to clinical pneumonia
Source: Sci Rep. 2017 Jul 25;7:6441. doi: 10.1038/s41598-017-06739-2 (PMC5527024; doi:10.1038/s41598-017-06739-2)
Supplement: Supplementary file 1 — Supplemental Info 1 [file 41598_2017_6739_MOESM1_ESM.doc]

**Title:**

**A rapid, low-cost, and microfluidic chip-based system for parallel identification of multiple pathogens related to clinical pneumonia**

**Author:**

Guoliang Huang^1*†^, Qin Huang^2*^, Lan Xie^1*^, Guangxin Xiang^2^, Lei Wang^2^,Hui Xu^2^, Li Ma^2^, Xianbo Luo^2^, Juan Xin^2^, Xinying Zhou^2^, Xiangyu Jin^1^, and Lei Zhang^2^

**Affiliations:**

^1^Department of Biomedical Engineering, the School of Medicine, Tsinghua University, Beijing 100084, China.

^2^National Engineering Research Center for Beijing Biochip Technology, Beijing 102206, China.

*These authors contributed equally to this work.

†Corresponding author. E-mail: [tshgl@tsinghua.edu.cn](mailto:tshgl@tsinghua.edu.cn)

**Supplemental Info 1.** Raw data of the 229 LRT discharge samples identified by the microfluidic chip based portable nucleic acid analyzer and ABI 7500 with 25-µL real-time PCR kits for *Sau*, MRSA, and *Mpn* detection at the same time.

| **SN** | **Sample No.** | **Sex** | **Age**  **(year)** | **Clinical Diagnostics** | **The results from ABI 7500 based PCR kits** | **The results from portable analyzer based microfluidic chip** | **The reanalysis by ABI 7500** | **The reanalysis by portable analyzer** |
| --- | --- | --- | --- | --- | --- | --- | --- | --- |
| 1 | R-001 | female | 29 | Pulmonary Infection | Mycoplasma Pneumonia | Mycoplasma Pneumonia |  |  |
| 2 | R-002 | female | 31 | Pulmonary Infection | Mycoplasma Pneumonia | Mycoplasma Pneumonia |  |  |
| 3 | R-003 | man | 85 | Pneumonia | MRSA, Staphylococcus Aures | MRSA, Staphylococcus Aures |  |  |
| 4 | R-004 | man | 85 | Pneumonia | MRSA, Staphylococcus Aures | MRSA, Staphylococcus Aures |  |  |
| 5 | R-005 | man | 18 | Pulmonary Infection | Mycoplasma Pneumonia | Mycoplasma Pneumonia |  |  |
| 6 | R-006 | man | 31 | Pulmonary Infection | Mycoplasma Pneumonia | Mycoplasma Pneumonia |  |  |
| 7 | R-007 | man | 6 | Pulmonary Infection | Mycoplasma Pneumonia | Mycoplasma Pneumonia |  |  |
| 8 | R-008 | man | 27 | Pulmonary Infection | Mycoplasma Pneumonia | Mycoplasma Pneumonia |  |  |
| 9 | R-009 | female | 26 | Pulmonary Infection | Mycoplasma Pneumonia | Mycoplasma Pneumonia |  |  |
| 10 | R-010 | man | 79 | Serious Pneumonia | MRSA, Staphylococcus Aures | MRSA, Staphylococcus Aures |  |  |
| 11 | R-011 | man | 79 | Serious Pneumonia | MRSA, Staphylococcus Aures | MRSA, Staphylococcus Aures |  |  |
| 12 | R-012 | female | 28 | Pneumonia | MRSA, Mycoplasma Pneumonia | MRSA, Mycoplasma Pneumonia |  |  |
| 13 | R-013 | female | 70 | Pneumonia | MRSA | MRSA |  |  |
| 14 | R-014 | man | 69 | Pneumonia | MRSA, Staphylococcus Aures | MRSA, Staphylococcus Aures |  |  |
| 15 | R-015 | female | 78 | Pneumonia | Mycoplasma Pneumonia | Mycoplasma Pneumonia |  |  |
| 16 | R-016 | man | 35 | Pneumonia | Mycoplasma Pneumonia | Mycoplasma Pneumonia |  |  |
| 17 | R-017 | female | 6 | Pneumonia | Mycoplasma Pneumonia | Mycoplasma Pneumonia |  |  |
| 18 | R-018 | man | 3 | Pneumonia | Mycoplasma Pneumonia | Mycoplasma Pneumonia |  |  |
| 19 | R-019 | female | 80 | Pulmonary Infection | Negative | Negative |  |  |
| 20 | R-020 | man | 48 | Pneumonia | Negative | Negative |  |  |
| 21 | R-021 | man | 79 | Pneumonia | MRSA, Staphylococcus Aures | MRSA, Staphylococcus Aures |  |  |
| 22 | R-022 | man | 75 | Pneumonia | Mycoplasma Pneumonia | Staphylococcus Aures | Mycoplasma Pneumonia | Staphylococcus Aures |
| 23 | R-023 | man | 48 | [Acute Bronchitis](http://www.baidu.com/link?url=4SoINzKqn1lIMyW--Atg4blS_D4Y2yBU7Xn2f_5CyJSy10_oGcEepJHr5Xra_wZTov7JofVl3_Wu6jf008DhPM6iLQONxlRyC4_9tEOJJMqevEKXVw0qFfnEF6dA2e5t) | Staphylococcus Aures | Staphylococcus Aures |  |  |
| 24 | R-024 | man | 55 | Pneumonia | MRSA, Staphylococcus Aures | MRSA, Staphylococcus Aures |  |  |
| 25 | R-025 | man | 46 | Pneumonia | MRSA | MRSA |  |  |
| 26 | R-026 | female | 72 | Pneumonia | MRSA, Staphylococcus Aures | MRSA, Staphylococcus Aures |  |  |
| 27 | R-027 | man | 82 | Pneumonia | MRSA, Staphylococcus Aures | MRSA, Staphylococcus Aures |  |  |
| 28 | R-028 | female | 54 | Pneumonia | MRSA | MRSA |  |  |
| 29 | R-029 | man | 80 | Pneumonia | MRSA, Staphylococcus Aures | MRSA, Staphylococcus Aures |  |  |
| 30 | R-030 | man | 62 | Acute Exacerbation Chronic Obstructive Pulmonary Disease | MRSA | MRSA |  |  |
| 31 | R-031 | man | 80 | Pneumonia | Staphylococcus Aures | Staphylococcus Aures |  |  |
| 32 | R-032 | man | 84 | Pneumonia | Staphylococcus Aures | MRSA, Staphylococcus Aures | Staphylococcus Aures | MRSA, Staphylococcus Aures |
| 33 | R-033 | man | 50 | Pneumonia | MRSA | MRSA |  |  |
| 34 | R-034 | man | 8 | Pneumonia | Negative | Negative |  |  |
| 35 | R-035 | female | 4 | Pneumonia | Negative | Negative |  |  |
| 36 | R-036 | man | 1 | Pneumonia | Negative | Negative |  |  |
| 37 | R-037 | man | 1 | Pneumonia | Negative | Negative |  |  |
| 38 | R-038 | female | 2 | Pneumonia | Negative | Negative |  |  |
| 39 | R-039 | man | 8 | Pneumonia | Negative | Negative |  |  |
| 40 | R-040 | man | 83 | Acute Exacerbation Chronic Obstructive Pulmonary Disease | Negative | Negative |  |  |
| 41 | R-041 | man | 50 | Pneumonia | Negative | Negative |  |  |
| 42 | R-042 | female | 44 | Pneumonia | Negative | Negative |  |  |
| 43 | R-043 | female | 67 | Pneumonia | Negative | Negative |  |  |
| 44 | R-044 | man | 77 | Pneumonia | Negative | Negative |  |  |
| 45 | R-045 | man | 74 | Pneumonia | Negative | Negative |  |  |
| 46 | R-046 | female | 29 | Pneumonia | Negative | Negative |  |  |
| 47 | R-047 | man | 69 | [Acute Bronchitis](http://www.baidu.com/link?url=4SoINzKqn1lIMyW--Atg4blS_D4Y2yBU7Xn2f_5CyJSy10_oGcEepJHr5Xra_wZTov7JofVl3_Wu6jf008DhPM6iLQONxlRyC4_9tEOJJMqevEKXVw0qFfnEF6dA2e5t) | Negative | Negative |  |  |
| 48 | R-048 | man | 73 | Pneumonia | Staphylococcus Aures | Staphylococcus Aures |  |  |
| 49 | R-049 | man | 72 | Pneumonia | Negative | Negative |  |  |
| 50 | R-050 | man | 78 | Pneumonia | Negative | Negative |  |  |
| 51 | R-051 | man | 83 | Pneumonia | Staphylococcus Aures | Staphylococcus Aures |  |  |
| 52 | R-052 | man | 76 | Pneumonia | Negative | Negative |  |  |
| 53 | R-053 | man | 64 | [Acute Bronchitis](http://www.baidu.com/link?url=4SoINzKqn1lIMyW--Atg4blS_D4Y2yBU7Xn2f_5CyJSy10_oGcEepJHr5Xra_wZTov7JofVl3_Wu6jf008DhPM6iLQONxlRyC4_9tEOJJMqevEKXVw0qFfnEF6dA2e5t) | Negative | Negative |  |  |
| 54 | R-054 | man | 65 | Acute Exacerbation Bronchiectasis | Negative | Negative |  |  |
| 55 | R-055 | man | 73 | Pneumonia | Negative | Negative |  |  |
| 56 | R-056 | female | 83 | Pneumonia | Negative | Negative |  |  |
| 57 | R-057 | man | 81 | Pneumonia | Negative | Negative |  |  |
| 58 | R-058 | female | 44 | Pneumonia | Negative | Negative |  |  |
| 59 | R-059 | man | 75 | Pneumonia | Staphylococcus Aures | Staphylococcus Aures |  |  |
| 60 | R-060 | female | 49 | Pneumonia | Negative | Negative |  |  |
| 61 | R-061 | man | 75 | Acute Exacerbation Chronic Obstructive Pulmonary Disease | Negative | Negative |  |  |
| 62 | R-062 | female | 94 | Pneumonia | Negative | Negative |  |  |
| 63 | R-063 | man | 57 | Acute Exacerbation Chronic Obstructive Pulmonary Disease | Negative | Negative |  |  |
| 64 | R-064 | man | 66 | [Acute Bronchitis](http://www.baidu.com/link?url=4SoINzKqn1lIMyW--Atg4blS_D4Y2yBU7Xn2f_5CyJSy10_oGcEepJHr5Xra_wZTov7JofVl3_Wu6jf008DhPM6iLQONxlRyC4_9tEOJJMqevEKXVw0qFfnEF6dA2e5t) | Negative | Negative |  |  |
| 65 | R-065 | female | 72 | Pneumonia | Negative | Negative |  |  |
| 66 | R-066 | man | 75 | Acute Exacerbation Bronchiectasis | Negative | Negative |  |  |
| 67 | R-067 | man | 72 | Pneumonia | Negative | Negative |  |  |
| 68 | R-068 | man | 76 | Acute Exacerbation Chronic Obstructive Pulmonary Disease | Negative | Negative |  |  |
| 69 | R-069 | female | 64 | Acute Exacerbation Chronic Obstructive Pulmonary Disease | Negative | Negative |  |  |
| 70 | R-070 | man | 21 | Acute Exacerbation Bronchiectasis | Negative | Negative |  |  |
| 71 | R-071 | man | 66 | Acute Exacerbation Chronic Obstructive Pulmonary Disease | Negative | Negative |  |  |
| 72 | R-072 | man | 70 | Pneumonia | Negative | Negative |  |  |
| 73 | R-073 | female | 73 | Pneumonia | Negative | Negative |  |  |
| 74 | R-074 | man | 60 | Pneumonia | Negative | Negative |  |  |
| 75 | R-075 | female | 82 | Acute Exacerbation Bronchiectasis | Negative | Negative |  |  |
| 76 | R-076 | man | 45 | Pneumonia | Negative | Negative |  |  |
| 77 | R-077 | man | 89 | Acute Exacerbation Chronic Obstructive Pulmonary Disease | Negative | Negative |  |  |
| 78 | R-078 | man | 85 | Acute Exacerbation Chronic Obstructive Pulmonary Disease | Negative | Negative |  |  |
| 79 | R-079 | female | 73 | Acute Exacerbation Chronic Obstructive Pulmonary Disease | Negative | Negative |  |  |
| 80 | R-080 | man | 79 | Pneumonia | Negative | Negative |  |  |
| 81 | R-081 | female | 43 | Pneumonia | Mycoplasma Pneumonia | Mycoplasma Pneumonia |  |  |
| 82 | R-082 | man | 79 | Acute Exacerbation Chronic Obstructive Pulmonary Disease | Negative | Negative |  |  |
| 83 | R-083 | man | 74 | Pneumonia | Negative | Negative |  |  |
| 84 | R-084 | man | 1 | Pneumonia | Negative | Negative |  |  |
| 85 | R-085 | man | 1 | Pneumonia | Negative | Negative |  |  |
| 86 | R-086 | man | 66 | Acute Exacerbation Chronic Obstructive Pulmonary Disease | Negative | Negative |  |  |
| 87 | R-087 | man | 83 | Pneumonia | Negative | Negative |  |  |
| 88 | R-088 | man | 79 | Pneumonia | Negative | Negative |  |  |
| 89 | R-089 | man | 58 | Pneumonia | Negative | Negative |  |  |
| 90 | R-090 | man | 68 | Pneumonia | Negative | Negative |  |  |
| 91 | R-091 | female | 79 | [Acute Bronchitis](http://www.baidu.com/link?url=4SoINzKqn1lIMyW--Atg4blS_D4Y2yBU7Xn2f_5CyJSy10_oGcEepJHr5Xra_wZTov7JofVl3_Wu6jf008DhPM6iLQONxlRyC4_9tEOJJMqevEKXVw0qFfnEF6dA2e5t) | Mycoplasma Pneumonia | Mycoplasma Pneumonia |  |  |
| 92 | R-092 | man | 86 | Pulmonary Infection | MRSA, Staphylococcus Aures | MRSA, Staphylococcus Aures |  |  |
| 93 | R-093 | man | 79 | Pneumonia | MRSA, Staphylococcus Aures | MRSA, Staphylococcus Aures |  |  |
| 94 | R-094 | man | 92 | Pulmonary Infection | MRSA, Staphylococcus Aures | MRSA, Staphylococcus Aures |  |  |
| 95 | R-095 | female | 61 | Pneumonia | Negative | Negative |  |  |
| 96 | R-096 | man | 60 | Pulmonary Infection | MRSA, Staphylococcus Aures | MRSA, Staphylococcus Aures |  |  |
| 97 | R-097 | female | 84 | Pneumonia | MRSA, Staphylococcus Aures | MRSA, Staphylococcus Aures |  |  |
| 98 | R-098 | man | 82 | Pneumonia | MRSA, Staphylococcus Aures | MRSA, Staphylococcus Aures |  |  |
| 99 | R-099 | female | 91 | Pneumonia | Negative | Negative |  |  |
| 100 | R-100 | female | 71 | Pulmonary Infection | Negative | Negative |  |  |
| 101 | R-101 | female | 78 | Pulmonary Infection | Negative | Mycoplasma Pneumonia | Negative | Mycoplasma Pneumonia |
| 102 | R-102 | female | 80 | Pulmonary Infection | MRSA | MRSA |  |  |
| 103 | R-103 | female | 78 | Pneumonia | Negative | Negative |  |  |
| 104 | R-104 | man | 28 | Pulmonary Infection | Mycoplasma Pneumonia | Mycoplasma Pneumonia |  |  |
| 105 | R-105 | female | 83 | Pneumonia | Negative | Negative |  |  |
| 106 | R-106 | female | 35 | Pneumonia | Mycoplasma Pneumonia | Mycoplasma Pneumonia |  |  |
| 107 | R-107 | female | 18 | Pulmonary Infection | Mycoplasma Pneumonia | Mycoplasma Pneumonia |  |  |
| 108 | R-108 | female | 18 | Pulmonary Infection | Mycoplasma Pneumonia | Mycoplasma Pneumonia |  |  |
| 109 | R-109 | female | 18 | Pneumonia | Mycoplasma Pneumonia | Mycoplasma Pneumonia |  |  |
| 110 | R-110 | female | 72 | Pulmonary Infection | MRSA, Staphylococcus Aures | MRSA, Staphylococcus Aures |  |  |
| 111 | R-111 | man | 49 | Pulmonary Infection | Mycoplasma Pneumonia | Mycoplasma Pneumonia |  |  |
| 112 | R-112 | man | 84 | Pulmonary Infection | MRSA,Staphylococcus Aures | MRSA,Staphylococcus Aures |  |  |
| 123 | E-001 | female | 9 | Pneumonia | Mycoplasma Pneumonia | Mycoplasma Pneumonia |  |  |
| 114 | E-002 | female | 7 | Pneumonia | Mycoplasma Pneumonia | Mycoplasma Pneumonia |  |  |
| 115 | E-003 | female | 2 | Pneumonia | Negative | Negative |  |  |
| 116 | E-004 | man | 6 | Pneumonia | Staphylococcus Aures | Staphylococcus Aures |  |  |
| 117 | E-005 | female | 1 | Pneumonia | Mycoplasma Pneumonia | Mycoplasma Pneumonia |  |  |
| 118 | E-006 | man | 10 | Pulmonary Infection | Negative | Negative |  |  |
| 119 | E-007 | female | 6 | Pneumonia | Mycoplasma Pneumonia | Mycoplasma Pneumonia |  |  |
| 120 | E-008 | man | 0.7 | Pulmonary Infection | Negative | Negative |  |  |
| 121 | E-009 | female | 2 | Pulmonary Infection | Mycoplasma Pneumonia | Mycoplasma Pneumonia |  |  |
| 122 | E-010 | man | 2 | Pulmonary Infection | Negative | Negative |  |  |
| 123 | E-011 | man | 6 | Pulmonary Infection | Mycoplasma Pneumonia | Mycoplasma Pneumonia |  |  |
| 124 | E-012 | man | 0.8 | Pneumonia | Negative | Negative |  |  |
| 125 | E-013 | female | 3 | Pneumonia | Negative | Negative |  |  |
| 126 | E-014 | man | 4 | Pneumonia | Negative | Negative |  |  |
| 127 | E-015 | female | 2 | Pulmonary Infection | Negative | Negative |  |  |
| 128 | E-016 | female | 5 | Pulmonary Infection | Negative | Negative |  |  |
| 129 | E-017 | female | 7 | Pneumonia | Negative | Negative |  |  |
| 130 | E-018 | man | 10 | Pneumonia | Negative | Negative |  |  |
| 131 | E-019 | man | 13 | Pulmonary Infection | Negative | Negative |  |  |
| 132 | E-020 | man | 0.5 | Pneumonia | Negative | Negative |  |  |
| 133 | E-021 | female | 0.6 | Pulmonary Infection | Negative | Negative |  |  |
| 134 | E-022 | female | 12 | Pneumonia | Negative | Negative |  |  |
| 135 | E-023 | man | 4 | Pneumonia | Negative | Negative |  |  |
| 136 | E-024 | man | 4 | Pulmonary Infection | Negative | Negative |  |  |
| 137 | E-025 | man | 0.9 | Pneumonia | Negative | Negative |  |  |
| 138 | E-026 | female | 1 | Pulmonary Infection | Negative | Negative |  |  |
| 139 | E-027 | man | 2 | Pulmonary Infection | Negative | Negative |  |  |
| 140 | E-028 | female | 3 | Pulmonary Infection | Negative | Negative |  |  |
| 141 | E-029 | female | 1.5 | Pneumonia | Negative | Negative |  |  |
| 142 | E-030 | female | 3 | Pneumonia | Negative | Negative |  |  |
| 143 | E-031 | female | 6 | Pneumonia | Negative | Negative |  |  |
| 144 | E-032 | female | 1 | Pulmonary Infection | Negative | Negative |  |  |
| 145 | E-033 | man | 11 | Pulmonary Infection | Negative | Negative |  |  |
| 146 | E-034 | female | 7 | Pneumonia | Mycoplasma Pneumonia | Mycoplasma Pneumonia |  |  |
| 147 | E-035 | female | 7 | Pulmonary Infection | Mycoplasma Pneumonia | Mycoplasma Pneumonia |  |  |
| 148 | E-036 | female | 8 | Pneumonia | Negative | Negative |  |  |
| 149 | E-037 | man | 10 | Pneumonia | Negative | Negative |  |  |
| 150 | E-038 | man | 1 | Pulmonary Infection | Negative | Negative |  |  |
| 151 | E-039 | man | 0.7 | Pneumonia | Negative | Negative |  |  |
| 152 | E-040 | female | 6 | Pulmonary Infection | Negative | Negative |  |  |
| 153 | E-041 | man | 11 | Pulmonary Infection | Negative | Negative |  |  |
| 154 | E-042 | female | 2 | Pulmonary Infection | Negative | Negative |  |  |
| 155 | E-043 | man | 12 | Pneumonia | Negative | Negative |  |  |
| 156 | E-044 | female | 17 | Pneumonia | Negative | Negative |  |  |
| 157 | E-045 | man | 8 | Pulmonary Infection | Negative | Negative |  |  |
| 158 | E-046 | female | 2 | Pneumonia | Negative | Negative |  |  |
| 159 | E-047 | man | 8 | Pneumonia | Negative | Negative |  |  |
| 160 | E-048 | man | 10 | Pneumonia | Negative | Negative |  |  |
| 161 | E-049 | man | 1 | Pneumonia | Negative | Negative |  |  |
| 162 | E-050 | female | 7 | Pulmonary Infection | Negative | Negative |  |  |
| 163 | E-051 | female | 3 | Pneumonia | Negative | Negative |  |  |
| 164 | E-052 | female | 1.1 | Pneumonia | Negative | Negative |  |  |
| 165 | E-053 | man | 10 | Pulmonary Infection | Negative | Negative |  |  |
| 166 | E-054 | man | 8 | Pulmonary Infection | Negative | Negative |  |  |
| 167 | E-055 | female | 1 | Pulmonary Infection | Negative | Negative |  |  |
| 168 | E-056 | man | 2 | Pulmonary Infection | Negative | Negative |  |  |
| 169 | E-057 | man | 2 | Pulmonary Infection | Negative | Negative |  |  |
| 170 | E-058 | female | 9 | Pneumonia | Negative | Negative |  |  |
| 171 | E-059 | man | 2.3 | Pulmonary Infection | Negative | Negative |  |  |
| 172 | E-060 | man | 2 | Pneumonia | Negative | Negative |  |  |
| 173 | E-061 | man | 4 | Pneumonia | Staphylococcus Aures | Staphylococcus Aures |  |  |
| 174 | E-062 | man | 0.4 | Catarrhal Pneumonia | Staphylococcus Aures | Staphylococcus Aures |  |  |
| 175 | E-063 | man | 0.5 | Catarrhal Pneumonia | Staphylococcus Aures | Staphylococcus Aures |  |  |
| 176 | E-064 | man | 1 | Pulmonary Infection | Negative | Negative |  |  |
| 177 | E-065 | female | 13 | Pneumonia | Negative | Negative |  |  |
| 178 | E-066 | female | 6 | Pulmonary Infection | Negative | Negative |  |  |
| 179 | E-067 | man | 0.75 | Pulmonary Infection | Negative | Negative |  |  |
| 180 | E-068 | man | 7 | Pneumonia | Negative | Negative |  |  |
| 181 | E-069 | man | 0.9 | Pneumonia | Negative | Negative |  |  |
| 182 | E-070 | man | 1.3 | Pulmonary Infection | Negative | Negative |  |  |
| 183 | E-071 | man | 0.6 | Pneumonia | Negative | Negative |  |  |
| 184 | E-072 | man | 1.2 | Pulmonary Infection | Negative | Negative |  |  |
| 185 | E-073 | female | 5 | Pneumonia | Negative | Negative |  |  |
| 186 | E-074 | man | 0.8 | Pulmonary Infection | Negative | Negative |  |  |
| 187 | E-075 | man | 1.5 | Pulmonary Infection | Negative | Negative |  |  |
| 188 | E-076 | man | 0.8 | Pulmonary Infection | Negative | Negative |  |  |
| 189 | E-077 | man | 3 | Pneumonia | Negative | Negative |  |  |
| 190 | E-078 | man | 1 | Pneumonia | Negative | Negative |  |  |
| 191 | E-079 | man | 1.5 | Pulmonary Infection | Negative | Negative |  |  |
| 192 | E-080 | female | 6 | Pneumonia | Mycoplasma Pneumonia | Mycoplasma Pneumonia |  |  |
| 193 | E-081 | female | 7 | Pneumonia | Negative | Negative |  |  |
| 194 | E-082 | man | 0.2 | Catarrhal Pneumonia | Negative | Negative |  |  |
| 195 | E-083 | man | 1.5 | Serious Pneumonia | Staphylococcus Aures | Staphylococcus Aures |  |  |
| 196 | E-084 | man | 0.9 | Serious Pneumonia | Mycoplasma Pneumonia | Mycoplasma Pneumonia |  |  |
| 197 | E-085 | man | 9.5 | Acute Pneumonia | Negative | Negative |  |  |
| 198 | E-086 | man | 0.8 | Serious Pneumonia | Negative | Negative |  |  |
| 199 | E-087 | man | 1.3 | Unresolved Pneumonia | Mycoplasma Pneumonia | Mycoplasma Pneumonia |  |  |
| 200 | E-088 | female | 2 | Pulmonary Infection | Negative | Negative |  |  |
| 201 | E-089 | man | 4 | Pneumonia | Negative | Negative |  |  |
| 202 | E-090 | female | 1.2 | Pulmonary Infection | Negative | Negative |  |  |
| 203 | E-091 | female | 10 | Pneumonia | Negative | Negative |  |  |
| 204 | E-092 | man | 1.3 | Serious Pneumonia | Negative | Negative |  |  |
| 205 | E-093 | man | 1.8 | Acute Pneumonia | Negative | Negative |  |  |
| 206 | E-094 | man | 4 | Pneumonia | Negative | Negative |  |  |
| 207 | E-095 | man | 0.1 | Chlamydia TrachomatisPneumonia | MRSA，Mycoplasma Pneumonia | MRSA | MRSA，Mycoplasma Pneumonia | MRSA |
| 208 | E-096 | man | 1.5 | Severe Fungal Pneumonia | Staphylococcus Aures | Staphylococcus Aures |  |  |
| 209 | E-097 | man | 1.5 | Pneumonia | Negative | Negative |  |  |
| 210 | E-098 | man | 6.5 | Pneumonia | Negative | Negative |  |  |
| 211 | E-099 | man | 6 | Pulmonary Infection | Staphylococcus Aures，Mycoplasma Pneumonia | Staphylococcus Aures，Mycoplasma Pneumonia |  |  |
| 212 | E-100 | man | 2 | Pneumonia | Negative | Negative |  |  |
| 213 | E-101 | man | 11 | Pulmonary Infection | Negative | Negative |  |  |
| 214 | E-102 | female | 6 | Pulmonary Infection | Negative | Negative |  |  |
| 215 | E-103 | man | 0.8 | Catarrhal Pneumonia | Negative | Negative |  |  |
| 216 | E-104 | man | 0.8 | Catarrhal Pneumonia | Negative | Negative |  |  |
| 217 | E-105 | man | 2.5 | Catarrhal Pneumonia | Staphylococcus Aures | Staphylococcus Aures |  |  |
| 218 | E-106 | man | 0.7 | Serious Pneumonia | Negative | Negative |  |  |
| 219 | E-107 | man | 0.8 | Catarrhal Pneumonia | Negative | Negative |  |  |
| 220 | E-108 | man | 0.1 | Serious Pneumonia | Negative | Negative |  |  |
| 221 | E-109 | man | 7 | Left side of Pneumonia | Negative | Negative |  |  |
| 222 | E-110 | man | 0.9 | Catarrhal Pneumonia | Negative | Negative |  |  |
| 223 | E-111 | man | 0.9 | Catarrhal Pneumonia | Negative | Negative |  |  |
| 224 | E-112 | man | 6 | Right side of Pneumonia | Negative | Negative |  |  |
| 225 | E-113 | man | 0.8 | Catarrhal Pneumonia | Staphylococcus Aures | Staphylococcus Aures |  |  |
| 226 | E-114 | man | 0.3 | Pneumonia | Staphylococcus Aures | Staphylococcus Aures |  |  |
| 227 | E-115 | female | 5 | Pulmonary Infection | MRSA，Mycoplasma Pneumonia | MRSA，Mycoplasma Pneumonia |  |  |
| 228 | E-116 | female | 0 | Pneumonia | MRSA | MRSA |  |  |
| 229 | E-117 | man | 1 | Pneumonia | Negative | Negative |  |  |
